# The prevalence and predictors of burnout among employees of the national emergency medical services system: evidence from Poland

**DOI:** 10.3389/fpubh.2026.1820538

**Published:** 2026-04-21

**Authors:** Mateusz Szczupak, Jacek Kobak, Anna Ingielewicz, Paulina Domańska-Gruszczyńska, Sabina Krupa-Nurcek

**Affiliations:** 1 Department of Anaesthesiology and Intensive Therapy of the Nicolaus Copernicus Hospital in Gdańsk, Gdańsk, Poland; 2 Department of Emergency Medicine of the Nicolaus Copernicus Hospital in Gdańsk, Gdańsk, Poland; 3 Department of Otolaryngology, Faculty of Medicine, Medical University of Gdańsk, Gdańsk, Poland; 4 Department of Otolaryngology, University Clinical Center, Gdańsk, Poland; 5 Department of Emergency Medicine, Faculty of Health Sciences, Medical University of Gdańsk, Gdansk, Poland; 6 Department of Emergency Medicine, St. Adalbert’s Hospital, Copernicus Healthcare Entity, Gdańsk, Poland; 7 Department of Surgery, Faculty of Medicine, Collegium Medicum, University of Rzeszów, Rzeszów, Poland

**Keywords:** burnout, emergency department, emergency medical services, healthcare workforce, occupational stress, Oldenburg Burnout Inventory

## Abstract

**Background:**

Burnout, classified in ICD-11 as a phenomenon associated with chronic workplace stress, represents a major challenge in emergency medicine. Employees of the State Emergency Medical System (SEMS) in Poland are exposed to high job demands, time pressure, traumatic events, and shift work, which may increase the risk of burnout. The aim of this study was to assess burnout severity and identify its determinants among SEMS employees using the Oldenburg Burnout Inventory (OLBI), within the framework of the Job Demands–Resources (JD-R) model and in accordance with STROBE recommendations.

**Methods:**

A cross-sectional study was conducted using an anonymous online survey between October 2025 and January 2026. Of 302 individuals who initiated participation, 261 completed the questionnaire (86.4% completion rate). Burnout was assessed in two dimensions: exhaustion and disengagement. Confirmatory factor analysis indicated limited separability between these dimensions. Due to non-normal data distribution, nonparametric tests (Mann–Whitney U and Kruskal–Wallis) were applied, with effect sizes reported. Multivariate regression analysis was performed to identify independent predictors of exhaustion, and k-means clustering was used to identify burnout profiles.

**Results:**

The study included 261 respondents (68.2% men, 31.8% women). Mean OLBI scores indicated a moderate level of burnout (disengagement: 2.508 ± 0.425; exhaustion: 2.657 ± 0.453), with exhaustion predominating. Salary satisfaction was significantly associated with both burnout dimensions (*p* < 0.001). Employees working in Emergency Departments reported higher exhaustion than those in Emergency Medical Services (*p* < 0.001). Multivariate analysis identified lower salary satisfaction, employment in Emergency Departments, and female gender as independent predictors of higher exhaustion. Cluster analysis revealed two distinct burnout profiles, with the high-burnout profile more frequent among Emergency Department staff and individuals reporting lower salary satisfaction.

**Conclusion:**

Moderate burnout, dominated by exhaustion, was observed among SEMS employees in Poland. Organizational factors, particularly salary satisfaction and workplace setting, were key correlates. These findings highlight the need for system-level interventions to improve working conditions and workforce sustainability, while acknowledging the cross-sectional design and psychometric limitations.

## 1 Introduction

Burnout is classified in the World Health Organization’s ICD-11 as a work-related phenomenon resulting from chronic occupational stress, defined by three components: energy depletion/feelings of exhaustion, increased psychological disengagement from work or negative/cynical attitudes toward work, and reduced sense of professional efficacy (1).^1^ This phenomenon is important not only for employee well-being but also for the quality of care and patient safety. A systematic literature review indicates that burnout among healthcare personnel is associated with poorer patient safety outcomes (2). Individuals suffering from burnout may experience negative health effects such as insomnia, depression, and hypertension (3–5).

Employees of the State Emergency Medical Services System are particularly vulnerable to the accumulation of occupational stressors: working under time pressure, exposure to traumatic events, unpredictable workload, the need to make critical decisions in the pre-hospital environment, and shift work. The State Emergency Medical Services system in Poland was established by the Act of September 8, 2006, to assist individuals facing sudden health threats. This act defines the principles of the system’s organization and operation.^2^ The State Emergency Medical Services System in Poland is a publicly funded, two-tier system comprising pre-hospital Emergency Medical Services teams and hospital-based Emergency Departments. EMS teams operate in the field and include paramedics, nurses, and physicians, typically working in shift-based systems (12- or 24-h shifts) and often under civil-law contracts. Emergency Departments, in turn, provide in-hospital acute care and are characterized by high patient turnover, overcrowding, and continuous workload. Employment conditions within SEMS are heterogeneous, including permanent employment contracts, civil law contracts, and mandate contracts, often combined with multi-site employment. Additionally, personnel may perform on-call duties or combine clinical work with administrative or teaching responsibilities. These organizational and contractual differences may influence workload, perceived job demands, and access to resources, and therefore represent relevant contextual factors in the assessment of occupational burnout.

Data from international studies suggest a significant prevalence of burnout among paramedics. A systematic review of studies on the incidence of burnout in this group revealed a wide range of results, depending, among other factors, on the tools used and the methodological quality of the studies (reported values across the included studies spanned a wide range). The authors of this review also emphasized the need for studies with strong methodological rigor, thereby providing reliable benchmarks for designing and evaluating preventive interventions (6).

In burnout research, selecting a measurement tool is crucial. The Oldenburg Burnout Inventory, developed by Demerouti et al. (7, 8), assesses burnout in terms of exhaustion and disengagement. Exhaustion refers to the feeling of physical and emotional fatigue, often experienced by emergency medical service workers due to demanding, emotionally taxing daily situations. Disengagement reflects a psychological distancing from work, potentially manifesting as cynicism or reduced involvement in professional relationships. A significant argument for using the OLBI in research conducted in Poland is the tool’s availability and the assessment of its psychometric properties in the Polish version. Validation studies have demonstrated satisfactory psychometric properties of the Polish adaptation of the OLBI and its applicability in Polish settings for measuring burnout (9). In recent years, studies on burnout among Polish paramedics using the OLBI have also been published. A pilot study published in 2024 in “Frontiers in Public Health” assessed burnout among paramedics in Poland using the OLBI, reporting the distribution of burnout levels in the study sample and analyzing factors associated with its dimensions (10). In parallel, other Polish empirical reports on burnout in this professional group are available, underscoring the problem’s relevance and importance among emergency medical services workers (11). At the same time, from the perspective of the State Emergency Medical System, it remains important to generate data that cover the broadest possible spectrum of professional roles and work environments within the SEMS, while accounting for potential organizational and sociodemographic factors.

## 2 Methods

### 2.1 Study design

A cross-sectional study was conducted to assess the severity of burnout among employees of the State Emergency Medical Services System in Poland, using the Oldenburg Burnout Inventory. The study aimed to provide a preliminary characterization of the magnitude of this phenomenon within the professional group under study and to identify factors potentially associated with specific dimensions of burnout. This study was conducted and reported in accordance with the STROBE guidelines for cross-sectional studies.

### 2.2 Characteristics of the research tool

The survey questionnaire used in the study was made available electronically via Google Forms and included socio-demographic questions and items from the Oldenburg Burnout Inventory (OLBI), enabling assessment of burnout levels among employees of the State Emergency Medical Services System in Poland.

The Oldenburg Burnout Inventory is a psychometric instrument comprising 16 items, eight for each of the two dimensions of burnout: exhaustion and disengagement from work. The questionnaire is based on a two-dimensional model of burnout, accounting for both the individual’s energy level and the degree of identification with their job, thereby allowing for a multifaceted interpretation of the results (7, 12).

The tool’s validation process, conducted among Dutch healthcare workers and administrative staff, including air traffic controllers, confirmed its high reliability. Cronbach’s alpha coefficient was 0.85 for both subscales, and factor analysis clearly confirmed the two-factor structure of the OLBI. The tool also demonstrated the ability to differentiate burnout levels across professional groups, indicating higher levels among healthcare workers than among air traffic controllers (7). The structural stability and psychometric properties of the OLBI were subsequently confirmed in numerous international studies (12). Responses are provided on a four-point Likert scale (1 – “agree,” 4 – “disagree”). Within each subscale, half the items are positive and half negative; negative items are recoded to ensure unidirectionality. Scores for the exhaustion and work disengagement subscales are calculated as the average of the individual item scores, ranging from 1 to 4, with higher scores indicating greater burnout symptoms. According to the authors’ recommendations, like the Maslach Burnout Inventory, a total burnout index is not calculated, and interpretation is performed separately for each subscale (7, 9, 13). Psychometric analyses confirmed good internal consistency and construct validity of the original version of the OLBI and its Polish adaptation, confirming the tool’s usefulness in scientific research (9, 13). The OLBI is less susceptible to response bias from the social desirability effect than the classic Maslach Inventory and enables the assessment of burnout in both clinical and general populations. Considering the dimension of disengagement from work is particularly important in professions with high emotional demands, such as emergency medicine. At the same time, the psychometric performance of the OLBI may vary across occupational groups and language adaptations. Therefore, although the OLBI remains a well-established instrument in burnout research, its dimensional structure and internal consistency should be verified within each studied population. For this reason, the present study evaluated not only burnout scores but also the internal reliability and factor structure of the OLBI in the investigated SEMS sample (14, 15).

The Oldenburg Burnout Inventory assesses two core dimensions: exhaustion and disengagement from work. In the present study, the term “disengagement” is used consistently to refer to the dimension reflecting psychological distancing and reduced involvement in work-related activities.

### 2.3 Data collection

Data were collected from October 1, 2025, to January 31, 2026, using an online survey questionnaire available to all study participants.

The survey was administered to employees of the State Emergency Medical Services in Poland. The questionnaire was distributed via online platforms, email, and social media links. The use of an online survey method enabled efficient and effective data collection from respondents from various regions of the country. The study was conducted in Polish.

This study used a non-probability convenience sampling strategy. The survey link was disseminated through professional online channels, e-mail distribution, and social media, and participation was voluntary. Therefore, a formal sampling frame covering the entire Polish State Emergency Medical Services workforce was not available, and the probability of inclusion for individual workers could not be determined. Therefore, the obtained sample should not be interpreted as statistically representative of all SEMS employees in Poland. Rather, it should be regarded as a heterogeneous national sample enabling the exploration of associations between burnout dimensions and selected occupational and organizational factors across major SEMS work settings.

Before completing the questionnaire, participants were briefed on the study’s purpose. After completing the main part of the survey, respondents were asked to provide basic demographic information. To ensure the anonymity and confidentiality of their responses, no personally identifiable information was collected. Participation in the study was voluntary, and informed consent was obtained by completing the survey, with the option to withdraw at any stage. Respondents were clearly informed of the anonymous and optional nature of their participation. The average time required to complete the entire questionnaire was approximately 15 min.

Given the exploratory nature of the study and the insufficient data to reliably estimate outcomes in the population of employees of the State Emergency Medical Services System, a formal sample size calculation was not performed. The sample size was determined by the availability of respondents and the recruitment period. A total of 302 individuals began the survey, of whom 261 completed it in full (completion rate = 86.4%). No *a priori* sample size calculation was performed because the study was exploratory, no reliable national reference estimates were available for the expected effect sizes in this target population, and the recruitment strategy was based on open voluntary participation rather than controlled enrolment. Nevertheless, the final analytical sample of 261 complete responses allowed stable estimation of descriptive statistics and nonparametric group comparisons, while multivariable modeling was restricted to a limited number of clinically and organizationally relevant predictors to reduce the risk of overfitting.

The study population was heterogeneous in terms of work organization, including differences in shift length (12-h, 24-h, or mixed systems), workload intensity, and workplace characteristics (pre-hospital EMS vs. hospital ED settings). These environments differ substantially in terms of patient volume, time pressure, and clinical complexity. Due to the observational design of the study, these factors were not standardized but were partially captured through variables such as workplace, work system, and number of working hours per month, which were subsequently included in statistical analyses.

In the absence of a national workforce registry available for direct analytical comparison, external validity should be interpreted with caution. The sample included the two principal operational settings of the Polish SEMS, namely Emergency Departments and pre-hospital Emergency Medical Services, and covered the main professional groups active within the system. However, because recruitment was voluntary and internet-based, the results should be generalized primarily to directional patterns and organizational correlates rather than to precise national prevalence estimates.

### 2.4 Inclusion and exclusion criteria

#### 2.4.1 Inclusion criteria

- Employment within the State Emergency Medical Services in Poland.
- Working as a system physician, system nurse, or paramedic.
- Active participation in professional work during the study period.
- Work experience of at least 6 months.
- Fluent knowledge of Polish, enabling independent completion of the survey questionnaire and informed consent to participate in the study.

#### 2.4.2 Exclusion criteria

- Not employed within the State Emergency Medical Services.
- Not working during the study period.
- Incomplete completion of the survey questionnaire.
- Work experience of less than 6 months.
- Language difficulties are preventing proper understanding of the survey questionnaire.

Only complete questionnaires were included in the final analysis. No missing data imputation procedures were applied.

### 2.5 Ethical considerations

In accordance with applicable Polish regulations governing non-interventional, anonymous, questionnaire-based studies that do not involve the collection of personal or sensitive medical data, formal bioethics committee approval was not required for this study. The study did not involve any experimental intervention, access to medical records, or collection of identifying information. Prior to participation, respondents were presented with information on the first page of the online questionnaire regarding the study aims, the voluntary nature of participation, the anonymity of responses, and the option to discontinue the survey at any time without consequences. Electronic informed consent was documented by the participant’s active progression to and completion of the questionnaire after reading this information. The study was conducted in accordance with the principles of the Declaration of Helsinki.

### 2.6 Statistical analysis

Statistical analysis was performed using nonparametric methods because the main outcome variables did not meet the assumptions of normality. Analyses were performed using Python 3.14.3.

Qualitative variables were presented as counts (n) and percentages (%). Quantitative variables were presented as means and standard deviations (SDs) or as medians and quartiles (Q1–Q3), depending on the distribution.

The Oldenburg Burnout Inventory (OLBI) consisted of 16 items rated on a 4-point Likert scale. Eight items were reverse-scored according to the original scoring procedure. Two subscales were calculated: disengagement (8 items) and exhaustion (8 items). Scale scores were expressed as the mean of the corresponding items, with higher values indicating higher levels of burnout. Internal reliability was assessed using Cronbach’s alpha for the total scale and for each subscale. Additional reliability analysis showed that removing selected items with low correlations with the total score increased Cronbach’s alpha to values above 0.70. However, to maintain consistency with the original OLBI structure and ensure comparability with previous studies, the full version of the instrument was used in the main analyses.

In addition, confirmatory factor analysis (CFA) was conducted to verify the two-factor structure of the OLBI (disengagement and exhaustion; 8 items per factor). CFA was performed using the semopy package in Python. Maximum likelihood estimation was applied. A model with two correlated latent factors was used; negatively worded items were recoded according to the original scoring procedure. Model fit was assessed based on χ^2^, CFI, TLI, and RMSEA. Model parameters were estimated using maximum likelihood. CFA was estimated using maximum likelihood, which should be interpreted cautiously in the context of ordinal Likert-scale responses. The CFA was included primarily to assess whether the expected two-factor structure could be replicated in this specific SEMS sample. Given the measurement level of the data, the results were treated as supportive rather than definitive psychometric evidence, and poor fit was interpreted conservatively. Future validation studies in this population should preferentially use estimation methods designed for ordinal indicators, such as weighted least squares with mean and variance adjustment. Nevertheless, this approach should be interpreted with caution, and future studies should consider estimation methods specifically designed for ordinal data (e.g., weighted least squares with mean and variance adjustment). Normality of distribution was assessed using the Shapiro–Wilk test. Due to significant deviations from normality in the disengagement and exhaustion subscales, nonparametric tests were used in comparative analyses.

Comparisons between two independent groups (e.g., workplace: ER vs. EMS; gender: women vs. men) were performed using the Mann–Whitney U test. Comparisons between more than two groups (occupation: physician, paramedic, nurse) were performed using the Kruskal-Wallis test. If the Kruskal-Wallis test was statistically significant, *post-hoc* analyses were performed using the Mann–Whitney U test with Holm’s correction for multiple comparisons.

Effect sizes were calculated to assess the strength of the observed differences. For the Mann–Whitney U test, the r coefficient was calculated from the standardized difference, whereas for the Kruskal-Wallis test, eta squared (η^2^) was calculated. The selection of variables for the multivariate regression model was based on a combined substantive and statistical approach. Variables showing an association with the outcome at *p* < 0.10 in univariate analyses were considered candidates for inclusion; however, final model specification also considered conceptual relevance within the Job Demands–Resources framework and the potential confounding role of key occupational characteristics. Accordingly, sex, profession, form of employment, work system, workplace, additional responsibilities, and exposure to violence/aggression were retained as covariates because they were considered theoretically plausible determinants or confounders of burnout, even when their univariate associations were weak. Multivariate regression analysis verified the basic assumptions of the linear model. The linearity of the relationship between the predictors and the dependent variable, the normality of the residual distribution (Shapiro–Wilk test and visual inspection of Q-Q plots), homoscedasticity (analysis of residual plots), and the absence of significant multicollinearity among the predictors were assessed. Multicollinearity was assessed using the Variance Inflation Factor (VIF), with VIF values < 5 considered acceptable. Model fit was assessed using the coefficient of determination (R^2^) and adjusted R^2^. Model diagnostics were examined using residual-versus-fitted plots to assess homoscedasticity, Q-Q plots and the Shapiro–Wilk test of residuals to assess approximate normality, and VIF statistics to assess collinearity. No major deviations indicating severe model misspecification were identified. Because the study was cross-sectional and exploratory, regression coefficients were interpreted as adjusted associations rather than causal effects. The level of statistical significance was set at *p* = 0.05. Given the heterogeneity of the study population, key professional and organizational variables (profession, employment status, work system, workplace, and working hours) were included in both univariate and multivariate analyses to account for potential confounding. This approach allowed the identification of independent predictors of burnout while minimizing bias arising from structural differences among professional subgroups.

To identify potential burnout profiles, a k-means clustering analysis was performed using two OLBI subscales (work disengagement and exhaustion). Variables were standardized prior to cluster analysis to ensure scale comparability. Solutions with 2 to 5 clusters were tested, and the optimal number of clusters was determined using the silhouette index. Cluster stability and internal validity were assessed using silhouette and internal consistency indices.

## 3 Results

The analysis included data from 261 respondents employed in Poland’s State Emergency Medical Services System. Detailed sociodemographic and professional characteristics of the study sample are presented in Table 1. Men predominated among the respondents (68.2%). The largest age group was 30–39 years old (43.3%). Most respondents had a master’s degree (68.6%). The occupational structure was dominated by system physicians (49.8%) and paramedics (46.7%). Over 67.05% of respondents were employed under civil law contracts, and 56.3% reported performing additional administrative or teaching duties. Most study participants (91.6%) reported experiencing violence or aggression from patients in the 3 months preceding the study, while 17.2% of respondents declared dissatisfaction with their remuneration.

**Table 1:** Sociodemographic characteristics of the study group.

Variable	Category	n	%
Sex	Male	178	68.2
	Female	83	31.8
Age (years)	<30	91	34.9
	30–39	113	43.3
	40–49	21	8.0
	50–59	34	13.0
	≥60	2	0.8
Education	Secondary education	18	6.9
	Higher bachelor’s degree	64	24.5
	Higher Master’s Degree	179	68.6
Profession in the State Emergency Medical Services System	Doctor	130	49.8
	Paramedic	122	46.7
	Nurse	9	3.4
Experience in the State Emergency Medical Services System (years)	<5	58	22.2
	5–10	84	32.2
	11–20	80	30.7
	>20	39	14.9
Form of employment	Employment contract	60	22.99
	Civil law contract	175	67.05
	Contract of mandate	26	9.96
Number of working hours per month	<160	34	13.0
	160–200	78	29.9
	201–240	83	31.8
	>240	66	25.3
Work system	Changes every 12 h	35	13.41
	Changes every 24 h	76	29.12
	Mixed system	150	57.47
Workplace	Emergency department	192	73.6
	Emergency Medical Services	69	26.4
Additional duties performed at the workplace	Yes	147	56.3
	No	114	43.7
Experience of violence from patients in the last 3 months of work	Yes	239	91.6
	No	22	8.4
Salary satisfaction	Very low	8	3.1
	Low	60	23.0
	Satisfactory	148	56.7
	High	33	12.6
	Very high	12	4.6

n, group size (number); %, percentage.

The internal reliability of the Oldenburg Burnout Inventory was assessed using Cronbach’s alpha (Table 2). For the work disengagement subscale, alpha = 0.583 was obtained, and for the exhaustion subscale, alpha = 0.661. For the OLBI total scale (16 items), Cronbach’s alpha was 0.765, indicating good internal consistency in the study sample.

**Table 2:** Internal reliability of the OLBI scale.

Scale	Number of items	n	Cronbach’s alpha
OLBI disengagement	8	261	0.583
OLBI exhaustion	8	261	0.661
OLBI total	16	261	0.765

n, group size (number).

Cronbach’s alpha for the disengagement subscale (*α* = 0.583) indicates moderate internal consistency in the study sample, lower than that reported in some validation studies. This may reflect the specificity of the studied professional population or the heterogeneity of the disengagement construct within the emergency medical services system’s work environment. Therefore, caution should be exercised in interpreting the results of this subscale.

To verify the factor structure of the Oldenburg Burnout Inventory, confirmatory factor analysis (CFA) was conducted on the classic two-factor model, including the dimensions of disengagement from work and professional exhaustion. The analysis was conducted on the full study sample (*n* = 261). Model fit indices are presented in Table 3.

**Table 3:** CFA—model fit indices.

Indicator	Value
n (to CFA)	261
χ^2^	603.52
df	103
p	<0.001
CFI	0.627
TLI	0.566
RMSEA	0.137

χ^2^, chi-square test statistic; df, degrees of freedom; p, *p*-value; CFI, Comparative Fit Index; TLI, Tucker–Lewis Index; RMSEA, Root Mean Square Error of Approximation; CFA, confirmatory factor analysis.

The obtained results indicated limited model fit to the empirical data. The chi-square test was statistically significant (χ^2^ = 603.52; df = 103; *p* < 0.001), suggesting the model deviated from the structure observed in the sample. The global fit indices were below recommended values: the Comparative Fit Index (CFI) was 0.627, and the Tucker–Lewis Index (TLI) was 0.566. The Root Mean Square Error of Approximation (RMSEA) reached 0.137, indicating poor model fit. The high correlation among the latent factors suggests a possible unidimensional structure of burnout in high-demand clinical environments, as reported in previous occupational studies. Despite generally unsatisfactory fit indices, most items demonstrated significant factor loadings in the predicted directions (Table 4). At the same time, a very high correlation was observed between disengagement and exhaustion, suggesting limited separability between these dimensions in the study population. These results may reflect the specific work environment within the State Emergency Medical Services System, in which burnout components may co-occur to a greater extent than in the general population. Additionally, an alternative one-factor structure was explored; however, it did not provide a clearly superior representation of the data compared with the original two-factor solution. Taken together, these findings suggest that the OLBI dimensions may be only partially separable in this population, and that burnout in SEMS personnel may manifest as a highly overlapping syndrome dominated by exhaustion.

**Table 4:** Standardized OLBI factor loadings in CFA analysis.

Content of the item	Standardized factor loading	*p*
Exhaustion		
Fatigue before finishing work	0.46	<0.001
The need for rest after work	0.63	<0.001
Good tolerance of work pressure (R)	0.49	<0.001
Emotional exhaustion from work	0.72	<0.001
Energy after work (R)	0.59	<0.001
Feeling worn out after work	0.77	<0.001
Coping with workload (R)	0.09	0.21
Energy at work (R)	0.18	0.01
Disengagement		
Finding new aspects of work (R)	0.41	<0.001
Negative statements about work	0.73	<0.001
Automatic execution of work	0.45	<0.001
Positive challenges (R)	0.43	<0.001
Gaining some distance from work	0.05	0.42
Negative feelings toward work	0.71	<0.001
The only kind of work (R)	0.30	<0.001
Work Commitment (R)	0.53	<0.001

All values are presented as standardized factor loadings. Items marked (R) were reverse-scored according to the original scoring procedure. Items were assigned to factors according to the classic two-factor structure of the OLBI.

Given the low internal consistency of the disengagement subscale and the poor fit of the two-factor CFA model, all findings concerning disengagement should be interpreted with caution. In addition to the main analyses based on the original OLBI structure, supplementary sensitivity checks were performed at the level of internal consistency. These checks showed that excluding items with the weakest item-total correlations improved alpha coefficients; however, because such modifications would alter the instrument’s original content domain of the instrument and reduce comparability with prior OLBI-based studies, the prespecified full-item version was retained for the primary analyses. For this reason, the most robust inferences from the present study are those related to exhaustion and the overall pattern of burnout burden, rather than to fine distinctions within the disengagement construct.

The results obtained in the Oldenburg Burnout Inventory are presented in Table 5. The mean value for the dimension of disengagement from work (OLBI disengagement) was 2.508 ± 0.425, while for the dimension of exhaustion (OLBI exhaustion) it was 2.657 ± 0.453. Analysis of medians and interquartile ranges indicated a moderate severity of both burnout dimensions in the study sample.

**Table 5:** Results obtained in OLBI.

Scale	n	Mean	SD	Median	Q1	Q3	Min	Max
OLBI disengagement	261	2.508	0.425	2.500	2.250	2.750	1.500	4.000
OLBI exhaustion	261	2.657	0.453	2.625	2.375	3.000	1.375	3.875
OLBI total	261	2.583	0.393	2.563	2.313	2.813	1.438	3.688

OLBI, Oldenburg Burnout Inventory; SD, standard deviation; Q1, first quartile; Q3, third quartile.

The assessment of normality using the Shapiro–Wilk test showed statistically significant deviations from a normal distribution for the disengagement subscale (W = 0.9869; *p* = 0.0176) and the exhaustion subscale (W = 0.9890; *p* = 0.0452), whereas no significant deviation was observed for the OLBI total score (W = 0.9938; *p* = 0.3590). Given the ordinal origin of the scale scores and the evidence of non-normality in both subscales, nonparametric methods were used for the principal comparative analyses (Table 6).

**Table 6:** Shapiro–Wilk test for the distribution of OLBI scores.

Scale	n	W	*p*
OLBI disengagement	261	0.9869	0.0176
OLBI exhaustion	261	0.9890	0.0452
OLBI total	261	0.9938	0.3590

n, group size (number); W, Shapiro–Wilk test statistic; p, *p*-value.

The results of univariate analyses of the relationship between burnout level and sociodemographic and professional variables are presented in Table 7. The analyses were conducted separately for the two dimensions of burnout assessed using the Oldenburg Burnout Inventory, i.e., disengagement from work and professional exhaustion. With respect to the dimension of disengagement from work, no statistically significant differences were found depending on gender (*p* = 0.662; η^2^ = 0.000), age (*p* = 0.266; η^2^ = 0.009), level of education.

**Table 7:** Univariate comparisons of OLBI scores depending on sociodemographic and professional characteristics.

Variable	Test	p (OLBI disengagement)	Effect size	p (OLBI exhaustion)	Effect size
Sex	Kruskal–Wallis	0.876	η^2^ = 0.000	< 0.001	η^2^ = 0.042
Age	Kruskal–Wallis	0.240	η^2^ = 0.006	0.705	η^2^ = 0.000
Education	Kruskal–Wallis	0.222	η^2^ = 0.004	0.010	η^2^ = 0.028
Profession	Kruskal–Wallis	0.699	η^2^ = 0.000	< 0.001	η^2^ = 0.051
Length of work	Kruskal–Wallis	0.590	η^2^ = 0.000	0.004	η^2^ = 0.059
Form of employment	Kruskal–Wallis	0.848	η^2^ = 0,000	0.888	η^2^ = 0.000
Number of working hours per month	Kruskal–Wallis	0.082	η^2^ = 0.025	0.055	η^2^ = 0.030
Work system	Kruskal–Wallis	0.399	η^2^ = 0.000	0.965	η^2^ = 0.000
Place of work	Kruskal–Wallis	0.002	η^2^ = 0.032	< 0.001	η^2^ = 0.108
Additional duties	Mann–Whitney U	0.015	r = 0.151	0.418	r = 0.050
Experience of violence/aggression	Mann–Whitney U	0.222	r = 0.075	0.868	r = 0.010
Salary satisfaction	Kruskal–Wallis	<0.001	η^2^ = 0.119	< 0.001	η^2^ = 0.085

OLBI, Oldenburg Burnout Inventory; p, *p*-value; η^2^, effect size measure for the Kruskal–Walli’s test; r, measure of the effect size for the Mann–Whitney U test.

(*p* = 0.161; η^2^ = 0.006), profession performed in the State Emergency Medical Services System (*p* = 0.568; η^2^ = 0.000), length of service (*p* = 0.613; η^2^ = 0.000), primary form of employment (*p* = 0.211; η^2^ = 0.007), or work system (*p* = 0.294; η^2^ = 0.002). The variable “average number of work hours per month” only trended toward statistical significance (*p* = 0.058; η^2^ = 0.030) but did not reach the assumed significance threshold.

Statistically significant differences in work disengagement were found depending on the location of work (*p* = 0.007; η^2^ = 0.035), additional professional responsibilities (*p* = 0.022; *r* = 0.142), and level of salary satisfaction (*p* < 0.001; η^2^ = 0.104). The effect sizes for these relationships were small to moderate, indicating limited but significant strength. The strongest relationship with work disengagement was observed for salary satisfaction, with a systematic increase in disengagement intensity as satisfaction decreased.

In the case of professional exhaustion, the number of variables demonstrating statistically significant relationships was significantly greater. Significant differences in exhaustion levels were found depending on gender (*p* < 0.001; η^2^ = 0.042), education level (*p* = 0.010; η^2^ = 0.028), profession (*p* < 0.001; η^2^ = 0.051), length of service in the state emergency medical services (*p* = 0.004; η^2^ = 0.059), place of work (*p* < 0.001; η^2^ = 0.108), and salary satisfaction (*p* < 0.001; η^2^ = 0.085). No significant differences were found in the level of exhaustion based on age (*p* = 0.705; η^2^ = 0.000), employment status (*p* = 0.888; η^2^ = 0.000), work system (*p* = 0.965; η^2^ = 0.000), number of hours worked per month (*p* = 0.055; η^2^ = 0.030), or experience of violence/aggression (*p* = 0.868; *r* = 0.010). For these variables, effect sizes were small to moderate, suggesting that individual demographic and organizational factors are significantly associated with occupational exhaustion, but none predominantly explain this dimension of burnout. The strongest correlations with exhaustion were observed for work setting and salary satisfaction, with the effect of work setting slightly stronger (η^2^ = 0.108) than that of salary satisfaction (η^2^ = 0.085).

However, no statistically significant differences were found in the level of professional exhaustion depending on the work system (*p* = 0.947; η^2^ = 0.000) or on the reported experience of violence or aggression from patients in the last 3 months (*p* = 0.978; *r* = 0.002).

In summary, univariate analysis showed that salary satisfaction was the only variable significantly associated with both dimensions of burnout assessed using the OLBI, work disengagement, and exhaustion. Furthermore, the work setting was significantly associated with both dimensions of burnout, with the association stronger for professional exhaustion.

Oldenburg Burnout Inventory results were analyzed by work location, reporting values as median and interquartile range (Me [IQR]).

Among individuals employed in the emergency department (*n* = 192), the median disengagement from work was 2.50 [2.25–2.75], while the median exhaustion was 2.75 [2.50–3.13]. In comparison, the group working in emergency medical services (*n* = 69) had lower values for both subscales: disengagement 2.38 [2.13–2.63] and exhaustion 2.38 [2.13–2.63]. Higher median values for both disengagement and exhaustion were observed among Emergency Department staff than among Emergency Medical Services personnel.

Because the OLBI subscales did not meet the assumptions of normality, the Mann–Whitney U test was used to compare two independent groups. The analysis revealed significantly higher work disengagement scores in the ED group than in EMS (U = 8254.0; *p* = 0.00233), with a small effect size (*r* = 0.188). Even more pronounced differences were observed for occupational exhaustion: ED staff achieved significantly higher scores than EMS staff (U = 9507.5; *p* < 0.001), with a moderate effect size (*r* = 0.332). A similar pattern was observed for the OLBI total score, which was also significantly higher in the ED group (U = 9156.5; *p* < 0.001), with a small-to-moderate effect size (*r* = 0.291). Methodologically, the obtained effect sizes indicate that work location is statistically significantly associated with burnout severity, with this association being stronger for the exhaustion dimension than for work disengagement. Emergency Department staff demonstrated higher exhaustion and overall burnout scores compared to Emergency Medical Services personnel. Detailed descriptive statistics and test results are presented in Tables 8, 9.

**Table 8:** OLBI and the place of work.

Place of work	n	OLBI disengagement Me [IQR]	OLBI exhaustion Me [IQR]	OLBI total Me [IQR]
Emergency department	192	2.50 [2.25–2.75]	2.75 [2.50–3.13]	2.66 [2.38–2.94]
Emergency medical services	69	2.38 [2.13–2.63]	2.38 [2.13–2.56]	2.38 [2.25–2.56]

OLBI, Oldenburg Burnout Inventory; Me, median; n, group size (number); IQR, interquartile range (Q1–Q3).

**Table 9:** OLBI and the place of work—Mann–Whitney U Test.

Scale	U	*p*	*r*
OLBI disengagement	8254.0	< 0.001	0.188
OLBI exhaustion	9507.5	< 0.001	0.332
OLBI total	9156.5	< 0.001	0.291

U, Mann–Whitney U test statistic; p, *p*-value; r, effect size measure for the Mann–Whitney U test.

Univariate analysis revealed statistically significant differences in burnout levels, as assessed by the OLBI, across salary satisfactilevels on (Tables 10, 11). For disengagement from work, significant differences were found between the groups (H = 34.352; *p* < 0.001), with a small to moderate effect size (η^2^ = 0.119). Median values indicated a clear gradient—the highest intensity of disengagement was observed in the groups with very low satisfaction (Me 2.812 [2.594–3.375]) and low satisfaction (Me 2.625 [2.500–2.906]), while the lowest in the groups with high and very high satisfaction (Me 2.250 [2.000–2.625] and 2.125 [2.125–2.500], respectively). *Post-hoc* analysis confirmed that these differences were most pronounced in comparisons between the “Low” and “Satisfactory” categories, the “High” and “Very High” categories, and selected comparisons involving the “Very Low” category (Table 12). A similar pattern was observed for professional exhaustion (H = 25.846; *p* < 0.001; η^2^ = 0.085). The highest levels of exhaustion were observed in the very low (Me 3.062 [2.562–3.188]) and low (Me 2.875 [2.625–3.250]) satisfaction groups, while lower values were observed in the satisfactory and very high (Me 2.500) satisfaction groups. In post-hoc analyses, significant differences after Holm’s correction were found primarily for the “Low” vs. “Satisfactory” and “Low” vs. “High” comparisons, indicating that a shift from low to at least satisfactory satisfaction is associated with a significant decrease in exhaustion.

**Table 10:** Oldenburg burnout inventory results depending on the level of salary satisfaction.

Salary satisfaction	n	OLBI disengagement Me [Q1–Q3]	OLBI exhaustion Me [Q1–Q3]	OLBI total score Me [Q1–Q3]
Very low	8	2.812 [2.594–3.375]	3.062 [2.562–3.188]	2.938 [2.578–3.312]
Low	60	2.625 [2.500–2.906]	2.875 [2.625–3.250]	2.781 [2.500–3.016]
Satisfactory	148	2.500 [2.250–2.750]	2.500 [2.250–2.875]	2.500 [2.312–2.750]
High	33	2.250 [2.000–2.625]	2.625 [2.375–2.750]	2.438 [2.188–2.688]
Very high	12	2.125 [2.125–2.500]	2.500 [2.188–2.656]	2.375 [2.188–2.500]

OLBI, Oldenburg Burnout Inventory; Me, median; n, group size (number); IQR, interquartile range (Q1–Q3).

**Table 11:** OLBI and salary satisfaction—Kruskal–Wallis test results.

Scale	H	*p*	η^2^
OLBI disengagement	34.352	< 0.001	0.119
OLBI exhaustion	25.846	< 0.001	0.085
OLBI total score	33.551	< 0.001	0.115

OLBI, Oldenburg Burnout Inventory; H, Kruskal–Wallis test statistic; p, *p*-value; η^2^, effect size measure for the Kruskal–Wallis test.

**Table 12:** *Post-hoc* comparisons of the Oldenburg burnout inventory scores depending on the level of satisfaction with remuneration after the Kruskal-Wallis test.

Scale	Group 1	Group 2	p (Holm)	r
OLBI disengagement	Low	High	<0.001	0.443
	Low	Satisfactory	< 0.001	0.263
	Low	Very high	0.0076	0.387
	Very low	High	0.011	0.493
	Very low	Very high	0.018	0.656
	Very low	Satisfactory	0.026	0.223
OLBI exhaustion	Low	Satisfactory	< 0.001	0.310
	Low	High	< 0.001	0.399
OLBI total score	Low	Satisfactory	< 0.001	0.320
	Low	High	< 0.001	0.468
	Low	Very high	0.0169	0.362

Group 1, very low and low salary satisfaction; Group 2, satisfaction, high and very high salary satisfaction; U, Mann–Whitney U test statistic; p (Holm), *p*-value adjusted for multiple comparisons using the Holm correction; r, effect size for the Mann–Whitney U test.

The OLBI total score also differed significantly between salary satisfaction levels (H = 33.551; *p* < 0.001; η^2^ = 0.115). The highest values were observed in the very low and low satisfaction groups (Me = 2.938 and 2.781), while lower values were observed in the high and very high satisfaction groups (Me = 2.438 and 2.375). In post-hoc analyses, significant differences after Holm’s correction remained between “Low” satisfaction and “Satisfactory,” “High,” and “Very High,” indicating a consistent relationship between salary satisfaction and overall burnout severity.

Salary satisfaction significantly differentiates all three OLBI indices (in all cases *p* < 0.001). The effect sizes (η^2^ = 0.085–0.119) indicate a small to moderate effect; this means that salary satisfaction is a significant, consistent correlate of burnout, but does not explain it in a dominant way.

Multivariate regression analysis showed that, after adjustment for selected sociodemographic and occupational covariates, salary satisfaction, workplace, and sex were independently associated with occupational exhaustion. A one-level increase in salary satisfaction corresponded to a lower OLBI exhaustion score (B = −0.149; 95% CI: −0.226 to −0.071; *p* < 0.001). Working in a hospital Emergency Department, compared with pre-hospital Emergency Medical Services, was associated with a higher exhaustion score (B = 0.262; 95% CI: 0.107 to 0.417; *p* < 0.001). Female sex was also associated with higher exhaustion (B = 0.125; 95% CI: 0.004 to 0.246; *p* = 0.043). The remaining variables did not show independent associations with exhaustion in the adjusted model (all *p* > 0.05). Because of the cross-sectional design, these estimates should be interpreted as adjusted associations rather than directional or causal effects.

The workplace remained a significant independent predictor. Working in a hospital emergency department was associated with higher exhaustion than working on an emergency medical team, after controlling for other variables (B = 0.262; 95% CI: 0.107 to 0.417; *p* < 0.001). Sex also influenced exhaustion: women had higher OLBI exhaustion scores than men (B = 0.125; 95% CI: 0.004 to 0.246; *p* = 0.043). The remaining variables (occupation, employment status, work system, additional responsibilities, and self-reported violence/aggression) did not demonstrate an independent association with exhaustion in this model (*p* > 0.05). Detailed data are summarized and presented in Table 13.

**Table 13:** Multivariate regression for OLBI exhaustion.

Predictor	B	95% CI	*p*
Salary satisfaction	−0.149	−0.226 to −0.071	< 0.001
Place of work: ED vs. EMS	0.262	0.107 to 0.417	< 0.001
Sex: female vs. male	0.125	0.004 to 0.246	0.043
Profession: paramedic vs. doctor	−0.072	−0.216 to 0.072	0.330
Profession: nurse vs. doctor	−0.241	−0.681 to 0.199	0.282
Form of employment: civil law contract vs. employment contract	0.046	−0.085 to 0.177	0.491
Form of employment: civil law contract vs. mandate contract	0.037	−0.126 to 0.199	0.659
Work system: 12-h shifts vs. mixed system	0.069	−0.085 to 0.224	0.379
Work system: 24-h shifts vs. mixed system	0.026	−0.085 to 0.136	0.651
Additional Responsibilities: Yes vs. No	−0.043	−0.150 to 0.063	0.426
Violence/aggression (3 months): yes vs. no	0.101	−0.115 to 0.317	0.359

ED, Emergency Department; EMS, Emergency Medical Services; B, regression coefficient; 95% CI, 95% confidence interval; p, *p*-value.

The multivariate regression model for the dependent variable, OLBI exhaustion, was statistically significant (F-test for the overall model, *p* < 0.001) and accounted for a significant portion of the variance in the score (R^2^ = 0.203; adjusted R^2^ = 0.168). The diagnostic analysis revealed no significant violations of the linear model assumptions: the distribution of residuals did not differ significantly from normal, and the VIF values for all predictors remained below the adopted threshold of 5, indicating no significant multicollinearity.

The interaction model did not demonstrate a statistically significant interaction between workplace and pay satisfaction on OLBI exhaustion (B interaction = −0.104; 95% CI: −0.328 to 0.120; *p* = 0.365). This means that, at the formal level, there was no evidence that the relationship between pay satisfaction and exhaustion differs significantly between the Emergency Department and the Medical Emergency Services.

Simultaneously, slope estimates indicate a potentially stronger negative relationship in the Emergency Department (B ≈ −0.159) than in the Medical Emergency Services (B ≈ −0.055), with a wider confidence interval for the Medical Emergency Team, consistent with the smaller sample size. Therefore, when interpreting the results, it should be emphasized that the lack of significance of the interaction does not allow conclusions about the actual strengthening of the impact of workplace satisfaction, whereas the direction of the estimate may provide a basis for further analyses in a larger sample. Detailed data are presented in Table 14.

**Table 14:** Interaction between salary satisfaction and workplace, dependent variable: OLBI exhaustion.

Parameter	Interpretation	B	95% CI	*p*
Work satisfaction	Slope in EMS	−0.055	−0.262 to 0.152	0.605
Place of work: ED vs. EMS	Difference between ED and EMS at the lowest level of salary satisfaction	0.562	−0.111 to 1.236	0.102
Satisfaction × ED interaction	Difference in slope in the ED vs. EMS	−0.104	−0.328 to 0.120	0.365

ED, Emergency Department; EMS, Emergency Medical Services; B, regression coefficient; 95%CI, 95% confidence interval; p, *p*-value.

A k-means cluster analysis conducted on two OLBI dimensions (disengagement and exhaustion) identified two stable burnout profiles. Cluster 1 (*n* = 136) was characterized by lower values on both dimensions, whereas cluster 2 (*n* = 125) included individuals with significantly higher disengagement and exhaustion. Solutions from k = 2 to k = 5 were tested, and the selection of two clusters was justified by the highest silhouette index value (mean silhouette score = 0.412), indicating the best separation of clusters and their relative internal consistency. For k = 3, 4, and 5, the silhouette values were lower (0.356, 0.321, and 0.298, respectively), confirming the optimality of the two-cluster solution. The identified clusters were interpreted as profiles of lower and higher burnout severity.

The relationship between burnout profile membership, job location, and salary satisfaction was then assessed. A significant correlation was found between cluster and workplace (χ^2^ = 21.61; *p* < 0.001; Cramér’s V = 0.288), indicating a moderate relationship. The higher burnout profile (cluster 2) was significantly more common among individuals working in ED than in EMS. At the same time, cluster membership was significantly associated with salary satisfaction (χ^2^ = 22.88; *p* < 0.001; Cramér’s V = 0.296). In practice, this means that burnout profiles identified using the clustering method reflect not only differences in OLBI levels but also differences based on key organizational factors (e.g., workplace) and assessments of working conditions (e.g., salary satisfaction). Detailed data are presented in Tables 15, 16.

**Table 15:** Burnout profiles (k-means; 2 clusters)—characteristics of OLBI results.

Cluster	n	OLBI disengagement Me [Q1–Q3]	OLBI exhaustion Me [Q1–Q3]	OLBI total score Me [Q1–Q3]
1	136	2.25 [2.00–2.41]	2.38 [2.13–2.50]	2.31 [2.19–2.44]
2	125	2.75 [2.63–3.00]	3.00 [2.75–3.25]	2.88 [2.75–3.06]

Me, median; n, group size (number); IQR, interquartile range (Q1–Q3).

**Table 16:** Burnout clusters, workplace, and salary satisfaction.

Variable	Test	χ^2^	*p*	Cramér’s V
Workplace: ED vs. EMS	chi-square	21.61	< 0.001	0.288
Salary satisfaction	chi-square	22.88	< 0.001	0.296

χ^2^, chi-square test statistic; Cramér’s V, a measure of the strength of the relationship for contingency tables; p, *p*-value.

## 4 Discussion

A cross-sectional study among employees of the State Emergency Medical Service in Poland found moderate levels of both dimensions of burnout, as assessed with the Oldenburg Burnout Inventory, with a clear dominance of the exhaustion component over disengagement from work. This study provides novel insights by combining psychometric validation of the OLBI, cluster analysis, and multivariate modeling in a large sample of emergency care professionals. These results are consistent with the two-dimensional concept of burnout proposed by Demerouti et al., in which exhaustion is a central component of the response to chronic job demands, while disengagement serves as a secondary regulatory strategy (7, 8). Although some foundational references included in this study date back more than a decade (e.g., the original formulation of the Job Demands–Resources model and the development of the OLBI), they remain essential for conceptual and methodological consistency. To ensure contemporary relevance, recent empirical studies on burnout in emergency medical services and healthcare professionals have also been incorporated, reflecting current evidence and trends in occupational health research. The lower reliability of the disengagement subscale is consistent with previous reports indicating the heterogeneity of this psychological construct and variability in OLBI psychometric parameters across populations (16–19). Confirmatory factor analysis results indicated limited fit of the classic two-factor model to the data. The observed strong correlation between the dimensions and the varying factor loadings may suggest partial overlap between these constructs in environments with high job demands. This interpretation is consistent with the Job Demands–Resources model, which posits that, under conditions of chronic and intense job demands, exhaustion and disengagement may function as closely related adaptive mechanisms (8).

The strongest and most consistent predictor of job exhaustion in univariate and multivariate analyses was pay satisfaction. Higher satisfaction with pay was associated with a significant reduction in exhaustion, regardless of sociodemographic and organizational factors. This result highlights the key role of organizational resources in the JD-R model and suggests that perceived pay adequacy may serve as a significant buffer against the effects of high job demands (8).

The workplace was a significant factor differentiating burnout levels. Emergency department (ED) staff demonstrated higher levels of exhaustion and OLBI total scores than emergency medical services (EMS) staff. This result is consistent with previous studies indicating that cognitive overload, high decision-making pressure, and limited organizational resources in the ED environment are strong predictors of burnout (5, 20). Similar observations were reported in Polish studies, confirming the importance of organizational factors in the development of burnout among emergency medical service workers (1, 10, 11).

Comparison with international literature should also remain nuanced. Although many studies identify high-demand emergency and acute care settings as environments associated with greater burnout risk, the magnitude and pattern of associations vary across countries depending on staffing models, remuneration systems, role autonomy, workload indicators, and the instruments used to assess burnout. In particular, differences between pre-hospital and hospital-based emergency care are not entirely consistent across studies, suggesting that organizational context may be as important as the professional role itself. Therefore, our findings should be interpreted not as universally generalizable across all EMS systems, but as evidence situated within the structure and working conditions of the Polish SEMS.

In the present dataset, recent exposure to workplace violence or aggression was not independently associated with exhaustion in adjusted analyses. This finding should not be interpreted as evidence that violence is unimportant for staff well-being. First, the prevalence of reported violence/aggression was very high, which may have reduced variability and limited discriminatory power. Second, the measure used in this study captured exposure in a relatively broad dichotomous manner and did not account for frequency, severity, cumulative exposure, or psychological consequences. Third, the impact of violence may be mediated by other mechanisms, such as perceived organizational support, fear, accumulated emotional exhaustion over longer periods, or intention to leave employment, none of which were directly measured here. International literature generally supports workplace violence as a relevant occupational stressor; therefore, our non-significant result should be interpreted as context-specific and methodologically constrained rather than contradictory to the broader evidence base (21–25).

Cluster analysis revealed two stable burnout profiles, further confirming the heterogeneity of the study population. A profile with higher levels of both dimensions was more common among ED staff and those reporting lower salary satisfaction, reinforcing the importance of systemic and organizational factors as key determinants of burnout. The observed association between female sex and higher exhaustion requires cautious interpretation. This finding does not imply an intrinsic sex-based susceptibility to burnout; rather, it may reflect differences in role expectations, work–family strain, emotional labor, occupational positioning within emergency care teams, or unmeasured organizational exposures. International studies on gender and burnout in healthcare professionals remain heterogeneous, with some reporting higher exhaustion among women, whereas others show attenuated or context-dependent differences after adjustment for working conditions and job demands. In the present study, the association persisted after adjustment, but the effect size was modest, and residual confounding cannot be excluded. Therefore, this result should be viewed as hypothesis-generating and deserving of further investigation in studies with more detailed psychosocial and occupational measures.

These results have significant implications for healthcare policy. In the face of staff shortages and growing demands on the emergency medical services system, persistent exhaustion can lead to increased staff turnover, sick leave, and decreased organizational effectiveness. Interventions aimed at improving organizational resources, particularly remuneration and working conditions, in emergency departments may be a key element of a system-stabilization strategy. These observations are consistent with recent large-scale evidence syntheses indicating that occupational stress and burnout among emergency medical services personnel represent a systemic challenge affecting workforce sustainability and quality of care (26).

From a patient safety perspective, burnout extends beyond staff well-being. A meta-analysis by Garcia et al. demonstrated its association with poorer indicators of care quality and safety (2), and prospective studies indicate that high levels of burnout may predict long-term work disability and antidepressant treatment (3, 4). Consequently, the results of this study should be interpreted as a signal of potential threats to the healthcare system’s functioning. Future research should employ longitudinal designs, larger samples, and advanced psychometric modeling to better understand the structure and dynamics of burnout among Emergency Medical Service employees.

### 4.1 Strengths

The strengths of this study include a relatively large and diverse nationwide sample, the use of a validated measurement tool, consideration of various professional roles within the emergency medical services system, and the application of advanced statistical methods, including confirmatory factor analysis, multivariate regression, and cluster analysis.

### 4.2 Limitations of the study

The present study has several limitations. First, its cross-sectional design precludes temporal or causal inference; all identified relationships should be interpreted as associations only. Second, recruitment was based on voluntary participation in an online convenience sample, which introduces self-selection bias and limits representativeness. Third, all variables were based on self-report, making the findings potentially vulnerable to reporting and recall biases, as well as social desirability effects. Fourth, psychometric limitations of the OLBI in this specific sample must be acknowledged: the disengagement subscale showed weak internal consistency and the CFA results did not support a well-fitting two-factor structure, which weakens confidence in fine-grained interpretation of burnout dimensions, particularly disengagement. Fifth, the study was not preregistered, which increases the risk of analytical flexibility inherent in exploratory observational research. Sixth, although several occupational and organizational variables were included in the adjusted analyses, residual confounding cannot be ruled out, particularly with respect to unmeasured psychosocial, institutional, and workload-related factors. Finally, some subgroup sizes were uneven, especially the nurse subgroup, which limits the stability and interpretability of profession-specific comparisons.

### 4.3 Directions for future research and activities

Future longitudinal studies are warranted to assess the dynamics of burnout and identify predictive factors over time. It is also worthwhile to extend the analysis to include psychological variables (e.g., coping strategies, mental resilience) and organizational indicators (on-call staffing, number of interventions, system overload).

From a practical perspective, it is advisable to implement interventions to strengthen organizational resources in line with the JD-R model, including measures to enhance transparency and the adequacy of remuneration, optimize workloads, and provide psychological support for ED staff.

Given the documented link between burnout and patient safety, preventive measures should be incorporated into quality and safety policies within the emergency medical services system.

## 5 Conclusion

(a) A moderate burden of burnout symptoms was observed among employees of the Polish State Emergency Medical Services System, with exhaustion constituting the more prominent and analytically more robust dimension.
(b) Lower salary satisfaction was consistently associated with higher burnout scores, particularly with occupational exhaustion.
(c) Employment in Emergency Departments was associated with higher exhaustion than work in pre-hospital Emergency Medical Services.
(d) Burnout burden within the SEMS workforce appears heterogeneous, as reflected by the identification of two distinct symptom profiles.
(e) Given the cross-sectional design and psychometric limitations of the disengagement subscale, the findings should be interpreted cautiously; nevertheless, they support the view that organizational conditions are important correlates of burnout and should be considered in workforce and patient-safety policies.

## Data Availability

The original contributions presented in the study are included in the article/supplementary material, further inquiries can be directed to the corresponding author.

## Footnotes

1
https://www.who.int/news/item/28-05-2019-burn-out-an-occupational-phenomenon-international-classification-of-diseases

2
https://isap.sejm.gov.pl/isap.nsf/download.xsp/WDU20061911410/T/D20061410L.pdf

## Glossary

AIS: Athens Insomnia Scale
CFA: Confirmatory Factor Analysis
CFI: Comparative Fit Index
CI: Confidence Interval
ED: Emergency Department
EMS: Emergency Medical Services
H: Kruskal–Wallis Test Statistic
ICD-11: International Classification of Diseases, 11th Revision
IQR: Interquartile Range
JD-R: Job Demands–Resources Model
K-means: K-means Clustering Algorithm
Me: Median
n: Sample Size
OLBI: Oldenburg Burnout Inventory
p: *p*-value
Q1: First Quartile
Q3: Third Quartile
R: Reverse-scored Item
R^2^: Coefficient of Determination
RMSEA: Root Mean Square Error of Approximation
SD: Standard Deviation
SEMS: State Emergency Medical Services
TLI: Tucker–Lewis Index
U: Mann–Whitney U Test Statistic
VIF: Variance Inflation Factor
W: Shapiro–Wilk Test Statistic
η^2^: Effect Size Measure for Kruskal–Wallis Test
χ^2^: Chi-square Test Statistic
